# Vexas Syndrome Without Macrocytosis: A Case Highlighting an Expanding Clinical Phenotype and Diagnostic Delay

**DOI:** 10.7759/cureus.112356

**Published:** 2026-07-09

**Authors:** Hishaam A Yunas, Muhammad Sharif, Idris J Khan, Aadil J Hussain

**Affiliations:** 1 Rheumatology, Wye Valley NHS Trust, Hereford, GBR; 2 Rheumatology, King's College London, London, GBR

**Keywords:** autoinflammatory disease, genetic testing, normocytic anaemia, uba1, vexas syndrome

## Abstract

VEXAS (Vacuoles, E1 enzyme, X-linked, Autoinflammatory, Somatic) syndrome is a recently established adult-onset autoinflammatory disorder caused by somatic mutations in the ubiquitin-activating enzyme 1 (UBA1) gene. The condition predominantly affects men in later stages of life and is characterised by recurrent systemic inflammation, fever, pulmonary involvement, dermatological manifestations, haematological abnormalities, and frequent corticosteroid dependence. Due to its heterogeneous clinical presentation, diagnosis can often be delayed, effectively resulting in significant morbidity and mortality.

We report the case of a 78-year-old man who presented with recurrent episodes of high-grade fever, a widespread rash, pulmonary infiltrates, and repeated episodes initially diagnosed as periorbital cellulitis. Despite extensive investigations and multiple courses of antibiotics, no infective source was identified. The patient demonstrated recurrent inflammatory flares that responded to corticosteroids but relapsed during steroid tapering. Over the course of approximately one year, he developed progressive systemic inflammation, constitutional symptoms, pulmonary involvement, and normocytic normochromic anaemia. Following emerging recognition that macrocytosis may not be universally present in VEXAS syndrome, the diagnosis was considered and confirmed by bone marrow examination and genetic testing, demonstrating a pathogenic UBA1b mutation (p.Met41Val, c.121A>G). Unfortunately, the patient deteriorated rapidly and died before the diagnostic results became available.

This case highlights the diagnostic challenges associated with VEXAS syndrome, particularly in patients lacking classical haematological features such as macrocytosis. Increased awareness of the expanding phenotypic spectrum of VEXAS syndrome is essential to facilitate earlier diagnosis and timely multidisciplinary management.

## Introduction

VEXAS syndrome (Vacuoles, E1 enzyme, X-linked, Autoinflammatory, Somatic) is a progressive, potentially life-threatening, adult-onset autoinflammatory disorder first described by Beck et al. in 2020. They identified that the syndrome is driven by somatic mutations in the ubiquitin-activating enzyme 1 (UBA1) gene arising in haematopoietic stem and progenitor cells, producing a clonal haematopoiesis that predominantly affects the myeloid lineage and erythroid progenitors. These variants impair ubiquitin activation, setting off a chain of cellular stress and innate immune dysregulation that produces a mix of systemic inflammation and bone-marrow failure [1].

Patients with VEXAS syndrome typically present in later life with refractory systemic inflammation and haematologic abnormalities [1]. Recurrent fever, weight loss, pulmonary infiltrates, and ophthalmic involvement are common, as are cutaneous manifestations such as neutrophilic dermatoses, leukocytoclastic vasculitis, erythema nodosum-like lesions, and other inflammatory skin eruptions [2,3]. Other widely reported features include recurrent episodes of presumed infection, relapsing polychondritis, and venous thrombosis [3,4].

Haematological abnormalities are a central feature of the disease, with macrocytic anaemia traditionally regarded as one of the key diagnostic clues. However, more recent studies have demonstrated that mean corpuscular volume (MCV) may remain within the normal range in some patients, potentially contributing to delayed recognition [5]. Given the significant morbidity and mortality associated with VEXAS syndrome, prompt diagnosis is increasingly important, particularly in patients with unexplained steroid-dependent systemic inflammation.

We present the case of an elderly man with recurrent inflammatory episodes initially attributed to infection who was ultimately diagnosed with VEXAS syndrome following bone marrow biopsy and UBA1 mutation analysis.

## Case presentation

**Figure 1:**
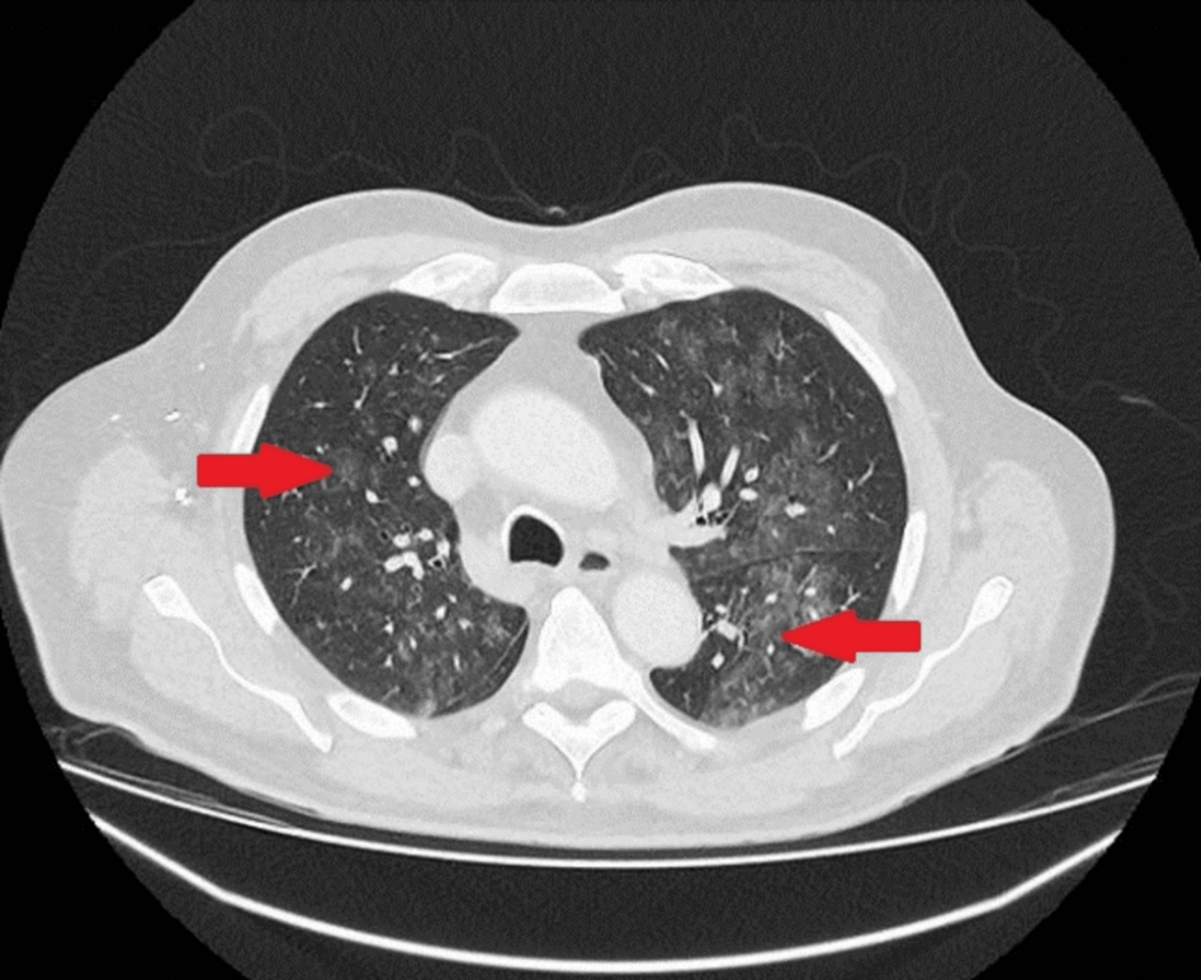
Computed tomography image from the first admission illustrating diffuse patchy and confluent areas of ground-glass haziness in all lobes of both lungs (red arrows), indicating infective/inflammatory aetiology.

A 78-year-old man was initially admitted in late 2024 with high-grade fever and shortness of breath. During admission, he developed a widespread skin rash. Computed tomography (CT) of the chest demonstrated inflammatory pulmonary changes (Figure 1), while inflammatory markers were markedly elevated (Table 1). He received several days of broad-spectrum antibiotics without significant clinical improvement, prompting rheumatology review.

**Table 1:** Relevant blood investigations throughout the patient’s diagnostic process. CRP: C-reactive protein; WCC: white cell count; Hb: haemoglobin; MCV: mean corpuscular volume.

Serum tests	Day 1	Day 23	Month 6	Month 7	Month 9	Month 10	Reference range
CRP	209	48	200	125	283	14	1-5 mg/L
WCC	5.6	7.1	5.9	4.8	15	5.7	4-10 x10^9^
Hb	132	89	100	103	108	92	130-170 g/L
MCV	88.7	92.1	92.7	93.9	86.4	93.3	83-101 fL
Platelets	290	295	190	210	384	271	150-410 x10^9^

Clinical assessment demonstrated a mixed dermatological picture consisting of vasculitic-type lesions together with lesions suggestive of erythema nodosum. An underlying inflammatory or autoinflammatory disorder was suspected. The patient was commenced on colchicine and corticosteroid therapy, with a marked clinical response. Fever, respiratory symptoms, and skin manifestations resolved, and he was subsequently discharged.

He was re-admitted with periorbital pain and swelling and was treated for presumed periorbital cellulitis. His symptoms improved following antibiotic therapy. Subsequent rheumatology follow-up resulted in initiation of methotrexate as a steroid-sparing agent while continuing prednisolone 10 mg daily.

Over the following months, he experienced recurrent admissions with similar episodes of presumed periorbital cellulitis. Methotrexate was discontinued because of concerns regarding recurrent infection. Although some episodes appeared to improve following a course of antibiotics, others demonstrated significant responsiveness to corticosteroid escalation. Prednisolone 10 mg daily was continued.

The patient subsequently developed recurrent episodes of high-grade fever without an identifiable source. During these admissions, he received empirical antibiotic therapy together with increased corticosteroid doses for presumed inflammatory disease flares. Attempts to taper corticosteroids below 15 mg daily consistently resulted in relapse, characterised by high-grade fevers, drenching night sweats, profound malaise, and elevated inflammatory markers.

Towards the end of 2025, he was admitted again with scrotal pain and swelling associated with productive cough and high-grade fever. At the time of admission, he was taking prednisolone 15 mg daily. Corticosteroid therapy was increased to 30 mg daily, and empirical antibiotic treatment was initiated.

Extensive investigations failed to identify an infective source. Repeated blood cultures and microbiological testing remained negative. Repeat CT imaging demonstrated persistent inflammatory pulmonary changes without evidence of focal infection. Transthoracic echocardiography, performed to rule out infective endocarditis, was unremarkable. Laboratory investigations revealed normocytic normochromic anaemia alongside persistently elevated inflammatory markers.

Despite escalation of prednisolone to 30 mg daily, the patient failed to demonstrate meaningful clinical improvement, and his condition progressively deteriorated. Around this time, emerging guidance and increasing recognition of VEXAS syndrome highlighted that affected patients may not invariably exhibit macrocytosis. Given the combination of recurrent steroid-dependent systemic inflammation, cutaneous vasculitic and erythema nodosum-like lesions, pulmonary involvement, constitutional symptoms, and unexplained anaemia, VEXAS syndrome was strongly suspected.

Following multidisciplinary discussion with haematology, a bone marrow biopsy was performed, and molecular genetic testing using next-generation sequencing (NGS) for VEXAS-associated UBA1 mutations was requested. Unfortunately, the patient’s clinical condition continued to worsen, and he died before diagnostic confirmation could be obtained. The results of the bone marrow biopsy (histopathology images unavailable) and genetic testing became available approximately three weeks after his death. The detailed cytomorphology of the bone marrow biopsy demonstrated trilineage haematopoiesis, and the genetic testing successfully identified the pathogenic p.Met41Val variant in the UBA1 gene, with variant allele frequency (VAF) of 64%. This result subsequently confirmed the diagnosis of VEXAS syndrome.

## Discussion

VEXAS syndrome is gaining increased recognition as an important cause of late-onset systemic inflammatory disease in older men. Since its initial description in 2020, a broad clinical spectrum has been identified, encompassing rheumatological, pulmonary, dermatological, and haematological manifestations [1,2].

This case demonstrates several characteristic features of VEXAS syndrome. Our patient experienced recurrent episodes of unexplained fever, constitutional symptoms, pulmonary inflammation, inflammatory skin lesions, and corticosteroid dependence. Repeated admissions were initially attributed to infection, including presumed periorbital cellulitis and fever of unknown origin. This is diagnostically challenging, as VEXAS syndrome frequently mimics severe infection due to the culmination of fever, elevated inflammatory markers, and radiological inflammatory changes [3].

Cutaneous manifestations were prominent in this case, including vasculitic lesions and erythema nodosum-like eruptions. Dermatological involvement has been reported in a majority of patients with VEXAS syndrome and may represent an early clue to diagnosis [2,3]. Similarly, pulmonary infiltrates are common and often contribute to recurrent hospitalisation [4].

A particularly notable feature of this case was the absence of macrocytic anaemia. While macrocytosis has traditionally been considered a key diagnostic hallmark, more recent reports in the literature have demonstrated that a normal MCV does not exclude the diagnosis [5]. Our patient developed normocytic normochromic anaemia, which may have contributed to delayed consideration of VEXAS syndrome. Increased awareness of this evolving phenotype is therefore essential.

Another characteristic feature was marked corticosteroid responsiveness, accompanied by an inability to successfully taper therapy. Steroid dependence is widely recognised in VEXAS syndrome and often results in prolonged exposure to high-dose corticosteroids with associated complications [2,4]. Conventional immunosuppressive agents such as methotrexate frequently demonstrate limited efficacy, as observed in this case.

The diagnosis of VEXAS syndrome requires demonstration of a somatic UBA1 mutation, usually following recognition of a compatible clinical phenotype. In Beck et al.’s 2020 study, three main UBA1 variants that result in a confirmed diagnosis of VEXAS syndrome were identified: p.Met41Leu (c.121A>C), p.Met41Val (c.121A>G), and p.Met41Thr (c.122T>C) [1]. Together, these account for around 95% of VEXAS-associated UBA1 variants described in the literature. UBA1b (the cytoplasmic isoform of UBA1) initiates the ubiquitin-proteasome system that is responsible for tagging damaged proteins for degradation. The Met41 somatic mutation disrupts production of the normal UBA1b protein, leading to translation starting at a different site (Met67), resulting in production of a shorter, catalytically impaired protein, UBA1c. This dysfunctional cytoplasmic isoform results in defective ubiquitin activation and dysregulated innate immune responses, as seen in VEXAS syndrome [2].

Using isoform-specific antibodies, it can be noted that whilst the p.Met41Leu and p.Met41Thr variants allow some residual translation of intact UBA1b, the p.Met41Val variant produces significantly less residual UBA1b relative to the others. p.Met41Val has been associated with a more severe phenotype, and the other two are associated with a less severe phenotype [2]. Notably, our patient was found to have the p.Met41Val variant in the genetic testing, and the severity of his symptoms correlates as expected with this genotype. Unfortunately, despite the eventual diagnostic confirmation, our patient died before the results became available. Although genetic testing may take several weeks to complete, with the diagnosis becoming delayed, particularly in rapidly progressive cases such as with our patient, consideration of its use earlier when suspecting VEXAS syndrome is key. Delving into VEXAS syndrome literature also exposes a question of whether patients with the p.Met41Val variant ought to be selected for allogeneic haematopoietic stem cell transplantation.

This case demonstrates the diagnostic complexity of VEXAS syndrome and highlights how the condition may masquerade as recurrent infection, inflammatory vasculitic disease, or fever of unknown origin. The absence of classical macrocytosis further complicated recognition and contributed to diagnostic delay. As awareness of the disease continues to evolve, clinicians should maintain a high index of suspicion in older male patients with unexplained systemic inflammation, characteristic dermatological manifestations, pulmonary involvement, corticosteroid dependence, and unexplained haematological abnormalities.

## Conclusions

VEXAS syndrome should be considered in older male patients presenting with recurrent steroid-responsive systemic inflammation, unexplained fevers, cutaneous vasculitic or neutrophilic manifestations, pulmonary inflammatory disease, and persistent elevation of inflammatory markers, particularly when extensive investigations fail to identify an infectious cause. This case highlights that normocytic anaemia identified on bloods should not exclude the diagnosis and may contribute to delayed recognition. Earlier consideration of VEXAS syndrome and prompt molecular testing for UBA1 mutations may facilitate timely diagnosis, reduce unnecessary antibiotic exposure, and allow earlier access to disease-directed therapies. Increased awareness of the expanding clinical spectrum of VEXAS syndrome remains essential to improving patient outcomes.
